# Accurate sound localization behavior in a gleaning bat, *Antrozous pallidus*

**DOI:** 10.1038/s41598-018-31606-z

**Published:** 2018-09-07

**Authors:** Dustin Brewton, Victoria Gutierrez, Khaleel A Razak

**Affiliations:** 1 Graduate Neuroscience Program, University of California, Riverside, USA; 2 Biology, University of California, Riverside, USA; 3 Department of Psychology, University of California, Riverside, USA

## Abstract

Acute auditory processing in bats is typically associated with echolocation. A subset of bats, called gleaners, listens to prey-generated noise to hunt surface-dwelling prey. Gleaners depend less on echolocation to hunt and, therefore, accurate localization of prey-generated noise is necessary for foraging success. Here we studied azimuth sound localization behavior in the pallid bat, a gleaning bat in which spatial encoding has been studied extensively. We tested pallid bats on a relatively difficult open loop task (single sound, duration ≤ 200 ms). The bats were trained to face the midline when stimulus was presented, and this was confirmed with video analysis. Bats localized broadband noise (5–30 kHz) from 1 out of 11 speakers spaced evenly across the horizontal plane of the frontal sound field. Approach to the correct speaker was rewarded. Pallid bats show accurate localization near the midline with mean errors between 3–6°. Remarkably, the accuracy does not decline significantly at peripheral locations with bats averaging  <~7° error upto 72° off midline. Manipulation of stimulus bandwidth shows that higher frequencies (20–30 kHz) are necessary for accurate localization. Comparative studies of gleaning bats will reveal convergent adaptations across auditory systems for non-echolocation-based behaviors in bats.

## Introduction

Sound localization is a primary function of the auditory system. The ability to localize sounds is important for successful foraging, avoiding predation, and locating a mate. Most bat species use echolocation for general orientation and foraging^1–5^. A small group of bat species have evolved another foraging strategy, called surface gleaning, in which prey-generated sounds are localized to hunt prey from a substrate (ground, foliage, etc.). The term ‘passive localization’ is used to describe the dependence of gleaning bats on prey-generated sounds to hunt. While gleaners can use a combination of passive localization, active echolocation and vision to hunt, the greater dependence on prey-generated noise distinguishes this group of bats from those that depend on echolocation for foraging^1,6–9^. Indeed, gleaning bats often reduce echolocation rates and intensities when closing in on prey^1,10,11^. Gleaning bats are present across multiple families, suggesting convergent evolution of this foraging strategy, but the neural specializations that support gleaning behavior across species are unclear. Given the dependence of gleaning bats on localizing relatively low intensity, prey-generated noise in the dark, it is likely that they have specializations for sound localization. However, few studies have examined the sound localization performance of gleaning bats. Here, we studied sound localization in the pallid bat (*Antrozous pallidus*), a gleaner, and report one of the most accurate localization performance across the frontal azimuth plane amongst vertebrates.

The pallid bat depends on prey-generated noise to hunt terrestrial prey such as crickets and scorpions^12,13^. Previous observations of sound localization behaviors in the pallid bat were performed in a manner that mimicked natural hunting behaviors wherein the bat was in flight or on a perch when the sound was presented^10,13^. Therefore, the head orientation in relation to the sound source was unknown. These studies reported localization accuracy of ~2–4°, but it remains unclear if and how the accuracy of localization is related to the eccentricity of source location. It is also unclear if properties of sound, such as duration and bandwidth show interactions with eccentricity of source location in determining accuracy. Therefore, it has not been possible to link behavioral outcomes to predictions from known cortical maps to examine the neuroethology of sound localization in the pallid bat.

Recent electrophysiological studies have suggested a cortical population code for the representation of 2D source locations^14–17^. The auditory cortex of the pallid bat contains a region selective for broadband noise with spectral energy present in prey-generated noise. Approximately a third of this noise-selective region contains neurons with peaked azimuth selectivity functions with preferred azimuth at ~0–15°. This suggests the presence of a cortical region specialized for midline azimuth localization. A second cluster within the noise-selective region contains neurons with sigmoid-shaped azimuth functions, with strong responses to contralateral locations. The intrinsic organization of this binaural cluster is such that the area of active cortex increases systematically as the sound moves from midline to more contralateral locations. Previous studies also indicated that azimuth selectivity is predicted by interaural intensity difference (IID) selectivity^16^. Ear directionality of the pallid bat is broad for frequencies below 15 kHz, and begins to sharpen and generate increasing IIDs for frequencies above 15 kHz^18^.

These studies make several predictions regarding azimuth localization by the pallid bat. First, the presence of a midline sensitive binaural cluster suggests that the bat should be most accurate near the midline. While this is not surprising given the comparative literature^19–23^, the electrophysiology data also suggests that the bat should be accurate at more eccentric locations. In the cluster of neurons with sigmoidal azimuth functions, the slopes of these functions are found across the frontal space, including peripheral locations. Slopes of approximately 40% of neurons course the midline. The remaining neurons have their azimuth slopes at more peripheral locations, including a sizable number with slopes located at >30° from midline^16^. The notion that azimuth function slopes are where neurons provide maximal information for spatial discrimination leads to the prediction that the bat should perform relatively accurately at peripheral locations. Ear directionality and IID/azimuth correlation results indicate that azimuth localization accuracy should improve with inclusion of frequencies >15 kHz^14,16,18^.

Here, we tested these predictions in a set up that allowed for the measurement of head position when a single, short noise burst was presented from 1 out of 11 speakers distributed evenly on the ground in front of the bat. This is an open loop task in that the sound was presented just once and with a duration that likely precluded head movements to update information. Thus, we tested the limits of azimuth localization accuracy in the pallid bat. The task for the bat was to localize the sound and crawl to the speaker for a reward. This allowed a measurement of absolute sound localization accuracy across frontal space.

## Results

**Figure 1:**
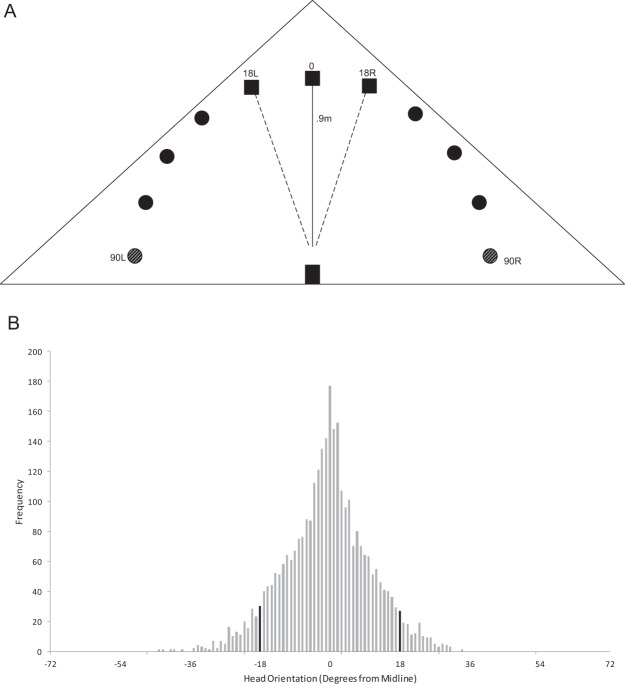
(**A**) A schematic of the speaker array used to test pallid bat azimuth localization accuracy. Eleven speakers (circles and boxes) were mounted (18^o^ separation between speakers) to sample across 180^o^ of frontal azimuth space. Bats were trained to crawl out of a cage (black box, 42 × 21 × 20 cm) and position themselves in a consistent location (grey box and asterisk). The bats were also trained to orient towards the midline (between the two dashed lines) for the stimulus to be presented. For two-way ANOVA analyses, speakers were grouped into midline (black squares) and peripheral (black circles) locations. The two most peripheral speakers (striped circles at 90^o^) were excluded from data analysis. (**B**) Distribution of measured head orientation relative to the midline. Data are separated into 1^o^ bins. Black bars mark the −18^o^ to 18^o^ window around the midline.

The main goal of this study was to quantify azimuth localization performance across the frontal hemifield and test the influence of duration and bandwidth on localization accuracy. Experiments were designed to identify potential interactions between location and duration or bandwidth in determining accuracy. Data were collected on an approach-to-speaker task (Fig. 1A). The 90° speaker data were not analyzed. The 90° positions represented the extremes of the array. Each of the 90° speakers only contained one adjacent speaker (the 72° location). This makes the 90° locations different from all other speakers, which contained two neighboring speakers. The 90° speakers were utilized in the study to maintain symmetry of speakers for the most eccentric locations (72°) that were quantified. Bats were trained to face the midline for sound presentation. The head position was analyzed off-line using video recordings to quantify the variation in head aim when sound was presented.

### Quantification of head orientation at sound presentation

We performed offline video analyses of head position to ensure that the bats were facing the midline speakers when the stimulus was presented. For this purpose, we randomly selected ~60% of all trials (3099/5164 trials) to quantify the bat’s head position in the video frame at which the sound was presented. In the vast majority of the trials (93%), the bat’s head aim was within ±18° of the midline at the time of sound presentation (Fig. 1B). There was no significant difference in the results if the 7% of trials for which the head aim was outside ±18° of the midline were included or not. Based on the observation that head position was within ±18° of the midline in 93% of the randomly chosen 3099 trials, we assume a similar distribution across all 5164 trials. Therefore, we present data from all 5164 trials below.

### Localization of Broadband Noise

**Figure 2:**
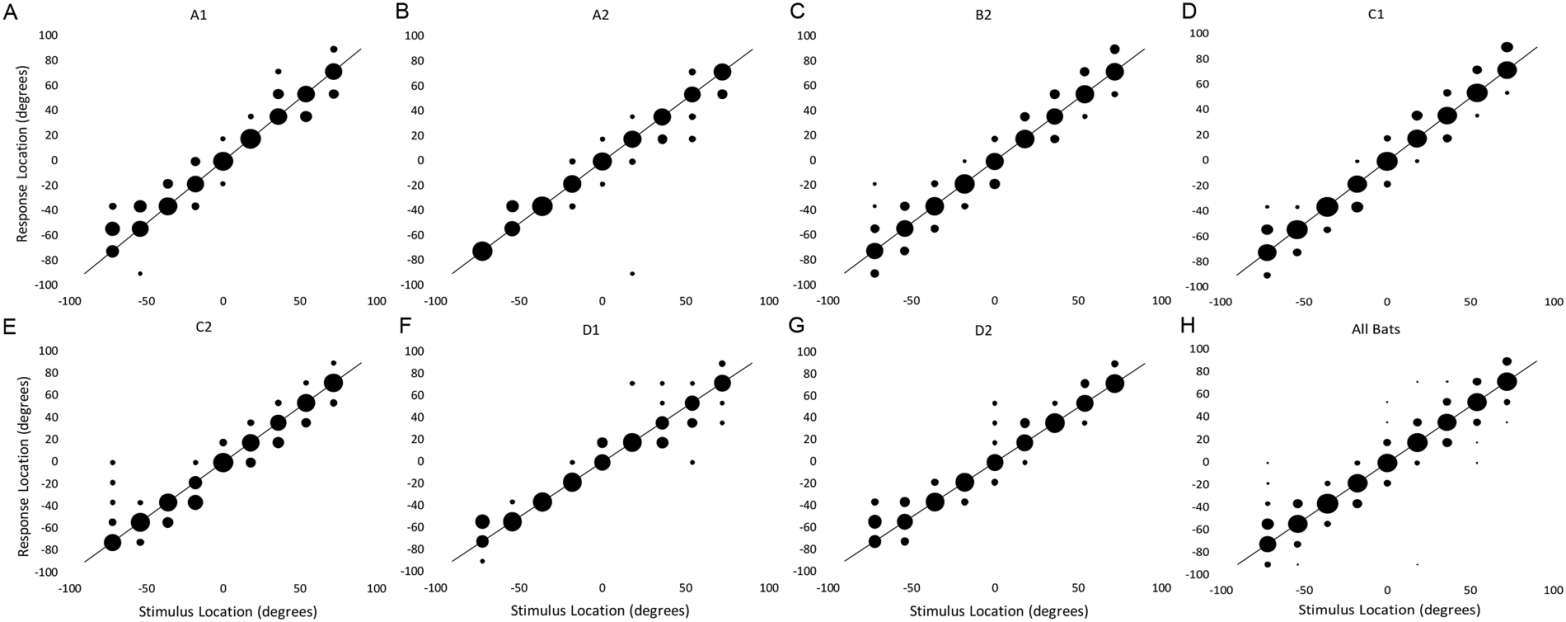
Confusion matrices demonstrate the performance of individual bats (**A–G**) and average performance (**H**). These data are for the 200 ms broadband noise stimulus. Circle position indicates the response location for a given stimulus location. The size of the circle indicates the percentage of trials on which the bat responded to a given speaker location. When a circle falls along the diagonal line, it is representative of 0° error. Circles falling above or below the diagonal by one position indicate 18° of error, and so on. A qualitative examination of the confusion matrices shows that the bats approached the correct speaker on the majority of trials, and mistakes were rarely more than one speaker away from the correct location.

Confusion matrices are often used to show sound localization accuracy^20,22,23^. The confusion matrix displays the actual location of the sound on the abscissa and the bat’s response location on the ordinate. The size of the circles indicates the percentage of trials in which the bat responded at a specific location. If the bat went to the correct speaker on every trial, all circles would be of maximum size and centered on the diagonal. A qualitative examination of each confusion matrix (Fig. 2A–G, each panel shows data from an individual bat, n = 7) shows that the bats were accurate to within one speaker location on most trials. This is further demonstrated by the composite performance matrix (Fig. 2H), which shows that on average, pallid bats rarely missed by more than 1 speaker away from the target.

**Figure 3:**
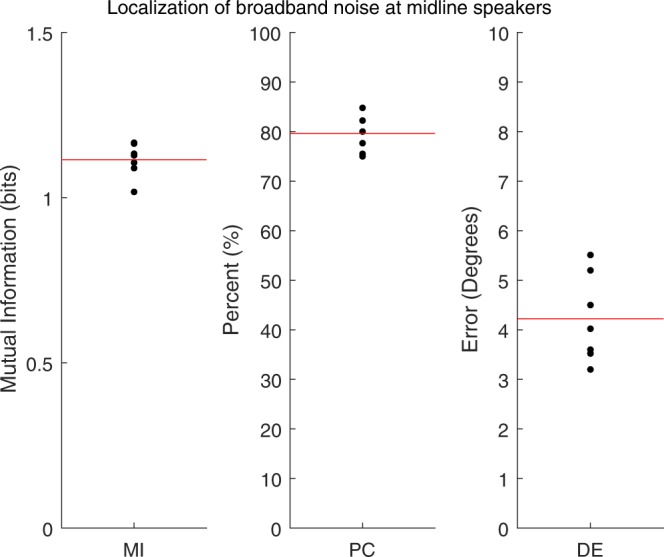
Performance of 7 pallid bats on the broadband noise (5–30 kHz, 200 ms) localization task, quantified using three separate measures: Mutual Information (**A**), Percent Correct (**B**), and Error (**C**). The solid red line in each plot indicates means of each measure. The theoretical maximum mutual information bit value is 1.59.

Three measures were calculated from confusion matrices to quantify sound localization accuracy: mutual information (MI), percent correct (PC), and degree error (DE) (Fig. 3). Localization of a 200 ms broadband noise (5–30 kHz) was quantified for the midline speaker (0°) and the two adjacent speakers (see Fig. 1, black squares for speaker locations). On average, pallid bats approached the correct speaker accurately ~80% of the time. The average localization error for these midline locations is ~4°. Mutual information is an additional measure that indicates performance accuracy. This value represents the predictability of unknown data (response location), given known data (stimulus location). This unit of information is measured in bits and is maximized by perfect performance. On average, the MI for broadband localization in pallid bats is 1.10 (maximum possible in this task is 1.59). Taken together, these data show that even with a relatively difficult open loop task, the pallid bat shows quite accurate localization of broadband noise near the midline.

### Localization accuracy does not deteriorate significantly, even at eccentric locations

Tables 1 and 2 show the performance of pallid bats localizing the 200 ms broadband noise across the entire speaker array. Localization is relatively accurate throughout the frontal azimuth plane. Comparison of performance from midline to peripheral space shows that the average percent correct changes from ~78% to 67% and the average degree error changes from ~4° to 7°. However, these trends were not statistically significant (One-Way Repeated Measures ANOVA: Percent Correct: F_(4, 7)_ = 1.490, p = 0.236; Error: F_(4, 7)_ = 1.795, p = 0.163), indicating that pallid bat sound localization ability does not decline even at relatively eccentric locations when localizing broadband noise. All subsequent analyses combine speakers into midline or peripheral groups (Fig. 1; filled boxes and circles, respectively) to determine interactions between the location and the duration or bandwidth of the stimulus.

**Table 1:** Percent of correct responses when localizing 200 ms broadband noise across the separate possible sound source locations of the speaker array are shown for each of the 7 tested bats.

Animal ID	Location (Degrees from Midline)				
	0	18	36	54	72
A1	87.50	79.31	72.00	63.64	52.94
A2	86.67	80.00	86.96	64.71	90.00
B2	67.65	83.33	68.85	68.85	66.18
C1	80.65	72.13	80.00	81.36	67.19
C2	87.50	55.88	65.71	77.42	74.19
D1	68.75	93.33	74.19	76.67	58.06
D2	70.59	78.13	90.00	65.63	63.33
**Means** ± **S.E**.	**78.47** ± **3.48**	**77.45** ± **4.34**	**76.82** ± **3.46**	**71.18** ± **2.70**	**67.41** ± **4.55**

Data are combined for each speaker equidistant to the left and right of the midline speaker. Therefore, data are approximately doubled for locations 18–72^o^ off midline, compared to the midline (0^o^). Means and standard error are reported in bold on the last row.

**Table 2:** Average error for all 7 bats across the speaker array.

Animal ID	Location (Degrees from Midline)				
	0	18	36	54	72
A1	2.25	3.72	5.76	7.09	9.53
A2	2.40	6.60	2.35	7.41	1.80
B2	5.82	3.00	5.61	5.61	6.88
C1	3.48	5.02	3.60	3.36	6.19
C2	2.25	7.94	6.17	4.06	8.13
D1	5.63	2.40	5.23	5.40	8.13
D2	8.47	3.94	1.80	6.19	7.80
**Means** ± **S.E**.	**4.33** ± **0.90**	**4.66** ± **0.75**	**4.36** ± **0.67**	**5.59** ± **0.56**	**6.92** ± **0.94**

All data are displayed as in Table 1.

### Influence of sound duration on azimuth localization

**Figure 4:**
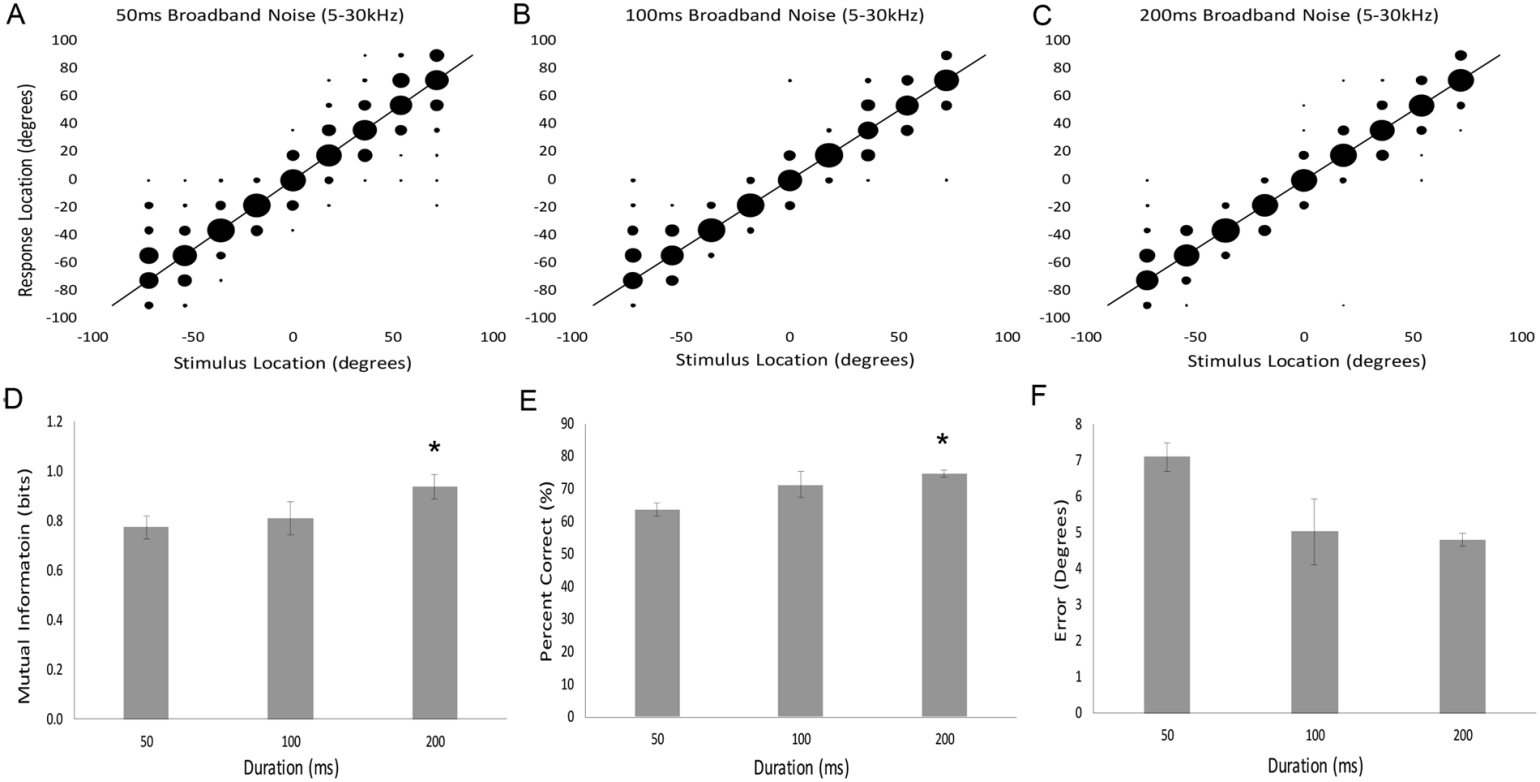
Effects of sound duration on localization behavior. Data are represented qualitatively in confusion matrices (**A**–**C**) and quantitatively in bar graphs (**D**–**F**). Bar graphs allow a comparison of group means (error bars denote s.e.) across the three different durations tested: 50, 100 and 200 ms. Asterisks indicate a significant difference from the 50 ms duration stimulus. *p < 0.05.

To determine if performance deteriorates for shorter sound durations^20–23^ in an azimuth dependent manner in the pallid bat, we tested two additional durations (50, 100 ms). Confusion matrices indicate better performance when the sound source duration is 200 ms compared to 50 ms (Fig. 4A–C). There is a main effect of sound source duration when measured by mutual information (Fig. 4D; F_(2, 4)_ = 7.295, p = 0.03) and the percent of correct responses (Fig. 4E; F_(2, 4)_ = 4.957, p = 0.05), but not when measured by degrees of error (Fig. 4F; F_(2, 4)_ = 4.066, p = 0.11). The mutual information between the perceived sound source and the location of the actual sound source is significantly greater when the sound source is 200 ms compared to 50 ms, but not 100 ms (Fig. 4D; *post-hoc*: 200 vs. 50 ms: p = 0.05, 200 vs. 100 ms: p = 0.20, 100 vs. 50 ms: p = 0.11). Post-hoc tests show comparable results for percent of correct trials (Fig. 4E; *post-hoc*: 200 vs. 50 ms: p = 0.01, 200 vs. 100 ms: p = 0.50, 100 vs. 50 ms: p = 0.15). There was no interaction between duration and location (two-way repeated measures ANOVA, F_(2, 4)_ = 4.066, p = 0.11), indicating that the longer duration sounds do not provide an advantage at specific locations. These data demonstrate that pallid bats are more accurate when localizing sounds of 200 and 100 ms duration, compared to 50 ms. While pallid bats miss more often when localizing 50 ms duration sounds, the performance is still quite accurate for these short duration sounds.

### Pallid bat azimuth localization accuracy improves when noise includes 20–30 kHz

**Figure 5:**
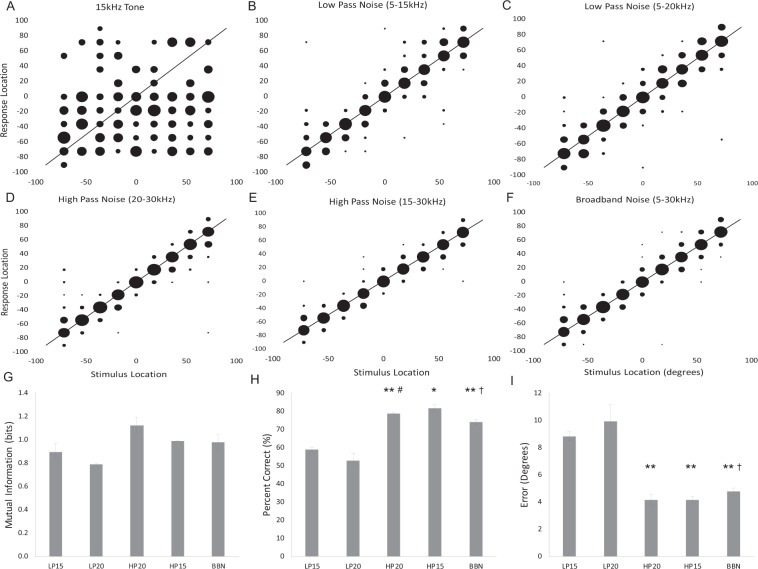
The presence of high frequencies in the stimulus is necessary for accurate localization performance. Data are represented qualitatively in confusion matrices (**A**–**F**) and quantitatively in bar graphs using the three data types: mutual information (**G**), percent of correct responses (**H**), and degrees of error (**I**). Bar graphs allow a comparison of group means (error bars denote s.e.) across the five different bandwidth conditions. Asterisks indicate a significant difference from the 15 kHz low-pass stimulus, pound signs indicate a significant difference from the 20 kHz low-pass stimulus, and crosses indicate a significant difference from the 20 kHz high-pass stimulus. *p < 0.05, **p < 0.01.

To test whether the bandwidth of the target sound affects azimuth localization performance, we first tested two pallid bats on their ability to localize a 15-kHz pure tone. While these bats approached a speaker in response to a sound presentation, the data show that they were unable to localize, or even lateralize, these sound sources. The bats showed a response bias toward the left side of the speaker array, regardless of the sound source (Fig. 5A). Due to the inability to localize pure tones by these two bats, we did not test additional bats and did not include tone localization performance in any further statistical comparisons.

Based on ear directionality studies that showed increasing IID values for frequencies >15 kHz, it has been hypothesized that the high frequencies in prey-generated noise would be necessary for accurate localization^18^. This hypothesis was supported (Fig. 5G–I). Confusion matrices demonstrate better performance for the HP noise (both 15 kHz HP and 20 kHz HP, termed HP15 and HP20, respectively) compared to LP noise (cut-off at 15 and 20 kHz, termed LP15 and LP20, respectively) across all locations (Fig. 5). Pallid bats demonstrated a significant increase in the percent of correct responses (Fig. 5H; F_(4, 4)_ = 98.599, p = 0.001), and a significant decrease in degrees of error (Fig. 5I; F_(4, 4)_ = 44.721, p = 0.001) for HP and broadband noise compared to LP noise. The percent of correct responses is significantly reduced for LP15 compared to the other sounds tested (Fig. 5H; *post-hoc*: LP15 vs. HP15: p = 0.03, LP15-HP20: p = 0.01, LP15 vs. BBN: p = 0.002). Post-hoc tests show a similar result for average error when comparing LP15 to the other sounds (Fig. 5I; *post-hoc*: LP15 vs. HP15: p = 0.01, LP15 vs. HP20: p = 0.01, LP15 vs. BBN: p = 0.01). When LP20 is compared to the HP and broadband noise, there is a trend for decreased accuracy (percentage correct, *post-hoc*: LP20 vs. HP15: p = 0.084, LP20 vs. HP20: p = 0.05, LP20 vs. BBN: p = 0.08; degree of error, *post-hoc*: LP20-HP15: p = 0.091, LP20-HP20: p = 0.109, LP20-BBN: p = 0.116), but no significant differences. When mutual information was used as measurement metric, there was no main effect of bandwidth, although there was a trend (Fig. 5G; F_(4, 4)_ = 4.440, p = 0.089). Additionally, there was no significant difference in performance when localizing HP15 noise compared to broadband noise on all 3 measurements (Mutual Information (MI): p = 0.92, Percent Correct (PC): p = 0.12, and Error (E): p = 0.12). Paradoxically, performance improved when localizing HP20 noise compared to broadband noise (MI: p = 0.03, PC: p = 0.03, E: p = 0.05). There was no interaction between bandwidth and location (two-way repeated measures ANOVA), indicating that these observations hold true across the entire frontal hemisphere. Taken together, these data demonstrate that the pallid bat performs better with azimuth localization when the noise stimulus is broadband and includes frequencies >15 kHz.

## Discussion

The pallid bat exhibits a localization accuracy of ~4° near the azimuth midline. Perhaps the more remarkable finding of this study is that the accuracy does not decline significantly at increasing azimuth angles, even up to 72° away from the midline. Most accurate localization was seen when frequencies >15 kHz were included in the stimulus, while the performance on the only pure tone tested was quite inaccurate.

### Comparison of azimuth localization across species

Humans, carnivores (cats and ferrets) and the barn owl have been best studied for absolute localization performance. The pallid bat is on par with these other vertebrates in midline localization. The performance of the bat at more peripheral locations is at least on par if not better (depending on the study) than the other animals tested. Comparison of sound localization estimates across species must consider the differences in task (pointing, approach, head orientation), stimulus used (tones versus noise), number of speakers tested and whether the task was absolute localization or relative localization (minimum audible angle). In general, localization is more accurate for broadband sounds than pure tones. Therefore, only studies that used broadband noise are compared here. One of the first assessments of human sound localization ability reported an average error of 4.6° when tasked with pointing to the location of a click^19^. A different study, using a head orientation task and a 150 ms, 1.8–16 kHz noise stimulus, reported <~8° error across the frontal hemifield, with the best performance near the midline (~2–3°)^21^. Although the standard deviation increased for more eccentric locations, the mean error was still <~8° even at 80° azimuth. Carlile and colleagues showed similar results in their study of human sound localization^24^.

In cats, the accuracy estimates vary considerably according to the outcome measure used. Localization measurements using head orientation showed errors <~5° in midline azimuth, but errors increase to ~30° for eccentric locations (e.g., 75°)^25^. Cats also exhibit increased errors at eccentric locations when localization of a 100 ms noise burst was measured with head orientation^26^. There are systematic undershoot errors in head orientation^25^ and saccades^27^ in cats and head orientation in ferrets^22^ for eccentric locations that may contribute to increased error in peripheral space. When minimum audible angles are used to measure spatial acuity, cats show excellent resolution (~4°) near the midline, which declines to ~9° in peripheral space^28^. When an approach to speaker task is used, the cat performs quite accurately^20,29–31^. The exact error calculations are not known from these papers, but the confusion matrices show performance akin to the pallid bat.

Ferrets show ~50–70% correct response on average when durations <200 ms were used in an approach to speaker task^22,32^. Performance is typically much better for midline locations compared to lateral locations. There is a considerable difference in percentage correct in approach to speaker tasks between the two carnivores tested, with the cat showing superior performance. Barn owls studied using head orienting behavior exhibit ~2° of error when localizing sounds near the midline. The performance drops off to between 6–10° at lateral azimuths^33^. The pallid bat data demonstrate that there is virtually no change in the percent of correct responses or mean error between 0° and 36° from the midline (−1.65% and +0.03°change, respectively). A significant change is not seen even when the sound is presented 72° away from the midline. When considering sound source locations that are in the range of 45–72° from the midline, pallid bats perform with accuracy better than or equal to human and barn owl localization and better than carnivores^21,33–35^. This is interesting when considering that the head size of the pallid bat is much smaller compared to the carnivores studied while using relatively similar frequency range (<35 kHz) for localization and points to both peripheral and central specializations that need to be further explored.

### Possible neural mechanisms of accurate azimuth localization behavior in the pallid bat

The auditory cortex is necessary for sound localization behavior in every species tested, particularly in approach-to-target tasks^20,30,36,37^. Previous electrophysiological studies of the pallid bat auditory cortex have led to the proposal of a population code for representation of 2D source locations^16,17^. Such a population code is found in the noise selective region, part of the primary auditory cortex of the pallid bat that represents frequencies between 5–35 kHz and is selective for broadband noise^14,38^. The noise selective region contains two clusters of neurons, distinguished by their interaural intensity difference (IID) and azimuth selectivity^14–16^. One cluster contains neurons with peaked IID selectivity, with best IID ~0 dB in most neurons. These neurons respond best to sources directly in front of the bat^15^.

The second cluster consists of neurons with sigmoidal IID selectivity functions (binaurally inhibited neurons). The binaurally inhibited neurons respond best to contralateral azimuth angles and show an inhibition of response as the source moves to relatively more ipsilateral loci^15^. The slope of the sigmoidal response can be centered at different azimuth locations (termed the 50% azimuth cutoff angle or simply ‘50% azimuth’). There is a diversity of 50% azimuth angles in the cluster of binaurally inhibited neurons. The topographical organization within this cluster depends on each neuron’s 50% azimuth angle. The organization is such that when sound moves from ipsilateral to contralateral locations, there is a systematic increase in the area of active cortex^16^. This idea is similar to hypotheses on spatial encoding in the inferior colliculus of the mustached bat^39^ and the superior colliculus of the cat^40^.

The accurate midline localization performance of the pallid bat may arise due to the over-representation of midline locations in the cluster of neurons with peaked IID selectivity. In addition, the forward facing large ears may enhance spatial sensitivity near the midline. The peaked cluster occupies ~25–30% of the noise selective region in the pallid bat auditory cortex. Similar midline preferring neurons are also seen in other mammals^41,42^ and may explain the generally better localization accuracy near midline compared to more eccentric locations across species. The main difference between the pallid bat and other species studied is the presence of a separate cluster of neurons whose response is maximal for sounds directly in front of the bat.

The accuracy of the pallid bat at eccentric sound locations may arise due to specializations in the cluster of binaurally inhibited neurons. First, there is a diversity of 50% azimuth in this cluster. Across neurons, these values cover a wide range of the contralateral hemifield. To the extent that the slopes of azimuth selectivity functions bear relevant localization information^43,44^, previous data from the cat cortex suggests that accurate midline performance arises through the preponderance of azimuth function slopes that transverse the midline^42^. A corollary of this hypothesis is the reduced performance expected at more eccentric locations because the sigmoidal functions tend to be flat (less informative) at increasingly contralateral locations. These predictions are met behaviorally in cats when tested with head orientation^25,26^. In the pallid bat, more neurons tend to have slopes between 0°–30°, but a substantial number of neurons have slopes between 30°–75°. Indeed, the area of active cortex within the cluster of binaurally inhibited neurons continues to increase linearly as the sound moves more from 30° to 60° azimuth and saturates past 60°. Thus, the activated area of this cluster contains information for azimuth discrimination even at peripheral locations^16^, and may explain the accurate performance of the bat at peripheral locations.

The fact that a number of cortical neurons have azimuth function slopes in peripheral space (>30°) is consistent with sharp changes in IID-azimuth relationships in lateral locations^18^. Neuronal IID responses are sensitive to such changes^15^. Ear directionality of the pallid bat is such that for frequencies <15 kHz, the maximum IID generated is ~10 dB. For frequencies >15 kHz, the ear becomes more directional and maximum IIDs increase up to 20–25 dB, particularly at peripheral azimuths. The rate of change of IIDs with azimuth increases with frequencies >15 kHz^18^. The availability of a broader range of IIDs at the higher frequencies likely explains the improved localization accuracy for sounds that include frequencies >15 kHz. Under free field conditions, only the high frequency tuned neurons will be sensitive to azimuth changes that generate IID changes in the 15–25 dB range. These neurons will be less activated by LP noise explaining the difference in performance for low-pass versus high-pass noise.

The bats were unable to localize a 15 kHz pure tone. In other mammals, reported accuracy of tone localization varies widely in the literature^19,20,45–49^. Given the description of the binaural clusters above, wherein the extent of active neurons represents locations, it is not surprising that the pallid bat performed poorly in tone localization tasks. In individual bats, 50% azimuth map in the cluster of binaurally inhibited neurons cuts across multiple isofrequency bands. When a narrowband sound is presented, only a part of the map will be activated and the spatial information will be ambiguous^50^. As the sound includes more frequencies, the full map can be utilized. It is unlikely the results are specific to the 15 kHz tone used here, because only part of the map will be activated at any single tone frequency used as stimulus.

### Methodological issues

A number of methodological issues need to be considered in interpreting the accuracy of sound localization behaviors reported here. We eliminated movement related cues from the mealworms by killing them first. To reduce the probability that the bats echolocated the worms, we provided dummy clay worms in non-target speakers. There was also a ~5 mm dip between the top of the platform and the wire mesh on which worms were placed reducing the probability that echolocation was used to detect worms from the bat’s starting position. We assume that the bats would have a more difficult time distinguishing a clay worm shape from a real worm, than a real worm from an empty speaker mesh. Moreover, trained bats rarely performed stop and search behaviors while approaching speakers.

These data were obtained with a relatively difficult open loop task in which the bats identified absolute location of a single, short duration (200 ms or less) stimulus. The latency from sound onset to the beginning of the head orienting behavior is unknown from this data set but has been estimated in other species. Cats demonstrate an orientation onset of ~50 ms following sound onset^51^, while big brown bat latency to head orienting stimuli is 60–100 ms^52^. Pinna movements likely have shorter latencies. Assuming the pallid bat head orienting response is similar, it is possible that the bats were able to update spatial information during presentation of the 100 and 200 ms duration sounds. The moderately improved performance for sounds with 200 ms duration compared to the 50 ms duration suggests that this may be the case. Alternately, the difference in performance between 50 and 200 ms could arise due to the different energy content of stimuli with different durations. We consider this unlikely because we randomly varied stimulus intensity between 60 and 70 dB SPL across trials, more than the 3–6 dB energy differences expected in stimuli with durations between 50–200 ms. The performance is only likely to improve if the sounds were longer or repeated allowing the bats to update information with pinna and head movements^21,25,26^. Given that the bat had to approach the target for reward, errors in remembering the location may also contribute to the overall accuracy.

The speakers in this study were separated by 18°. It is unclear if such quantization over- or under-estimates accuracy. On the one hand, *a priori* knowledge of speaker distribution may provide an advantage in localization. On the other, the accuracy may be an underestimate because even a single mistake by one speaker introduces an 18° error to the average error estimate. A previous study of the pallid bat sound localization was performed by dropping crickets on the floor^13^. While this approach avoided fixed speaker locations, the head position of the bats when crickets were dropped was not known, precluding a comparison of midline versus peripheral locations. Nevertheless, this study found an accuracy ~2–3° with this task. The sound durations of the stimuli were <25 ms. Here, we report accuracy ~4°. The relative consistency of the reported results, regardless of the differences in study design, suggest that quantization played a minimal role in error estimates reported here.

## Conclusions

The behavioral performance and the cortical mechanisms identified suggest that the specializations for sound localization in the pallid bat may provide insight into general mechanisms of mammalian sound localization. Comparative sound localization studies of gleaners will reveal constraints and adaptations in the auditory systems of bats to improve acuity in non-echolocation based behaviors. Despite the information that the binaurally inhibited cluster of neurons may provide at peripheral locations, the overall organization of the auditory cortex emphasizes midline azimuths. While we predicted accurate performance in the periphery, the similarity in peripheral and midline performance is a surprising finding. It is possible that, in a single source task in relatively quiet background conditions, the system was not pushed to reveal differential responses at midline and peripheral locations. Future experiments with masking will be performed to determine possible differences in midline and peripheral accuracy. Future studies will also examine the relative contributions of the two different binaural clusters to sound localization accuracy in the pallid bat using targeted chemical manipulations.

## Methods

### Animals

Pallid bats were netted in California, Arizona, and New Mexico and housed in an 11 × 14 feet flight room at the University of California, Riverside. The room was maintained on a reversed 12-hour light/dark cycle. Bats involved in the experiments were paired and housed in standard mouse cages with *ad libitum* access to water. The bats were moved to a different room (13 W × 14 L × 9 H feet) for training and testing. This room contained the speaker array at one corner and was anechoic (Sonex, 3in foam). The bats were returned to the flight room and left overnight in their cages at the end of trials each day. All experimental procedures used were approved by the University of California, Riverside Institutional Animal Care and Use Committee (IACUC, AUP20160044). All methods were performed in accordance with the IACUC guidelines and regulations. A total of 8 bats were used for this study. Bats were fed only in the room where testing occurred for the duration of the experiments. Weights of the bats were monitored daily and maintained above 80% of the starting weight.

### Apparatus

11 holes were cut out of a plywood board, distributed in a semicircular pattern with each hole separated by 18° from the next. The radius of the semicircle was 0.9 m. Speakers were mounted to the underside of each hole in the plywood board (Fig. 1A). The plywood board was placed horizontally on the floor such that the speakers faced upwards. A wire mesh was affixed on top of each hole to allow placement of freshly killed mealworms as reward for accurate localization. The wire mesh was ~5 mm below the surface of the platform. All non-target locations contained dummy mealworms made of clay and with similar dimensions as real worms. A 10.5 × 19 × 8in (L × W × H) box was secured to the middle of the semicircular array of speakers (Fostex FT17H), such that an opening at one end of the box was equidistant (0.9 m) from all speakers. The bat entered the speaker arena through this opening. Two infrared video cameras (XA10 Professional, Canon) were mounted directly above the opening in the box. One camera was angled such that the entire array was visible to analyze the approach behavior. The second camera was zoomed into the opening in the box from which the bats would initiate their localization trials. The recordings from this camera were used to analyze head position at sound presentation (see *Head Orientation Measurements*). All trials were recorded under infrared light illumination (IRLamp6; Bat Management IR Kit). The experimenter also wore a headlamp with red light to observe the bats during all trials.

### Sound localization training

Bats were conditioned to associate noise bursts with food reward. Pallid bats do not readily approach speakers generating noise^13^. Therefore, a specific sequence of training was used on each bat. To begin, each bat fed on freshly killed mealworms *ad libitum* for 30 minutes per day in the testing arena, until they became comfortable enough to remain in the testing arena voluntarily for the entire duration. No measures were taken to keep animals in the arena, and the bats could fly away at will. After a few days, the bats would remain foraging for the entire 30-minute duration. Mealworms were then moved to a random speaker location from which a broadband noise (5–30 kHz, 200 ms white noise) was repeated continuously at a rate of 1 Hz. Eventually, the bats would readily localize the sound, move to the speaker, and obtain food reward. At this point, only the sound-producing speaker contained a single mealworm, while all other speakers contained clay mealworm models.

To ensure relatively consistent head positions at the time of sound presentation, bats were trained to walk through a plastic box with holes cut out on each end to enter the arena before foraging. Sound presentation only began when the bat emerged from the box. The noise was played less frequently each day, until the bats attempted to localize only one sound presentation consistently. Bats were then trained to wait at the same location just outside the box with their heads facing forward until the sound was played. If they tried to approach a speaker or turned their heads off center, the bats were given a brief time out before being allowed to try the task again. A running average of degrees of error at the midline speakers over five days was calculated to determine when testing should begin. A correct response is counted as an error = 0°. If the bat missed the target by one speaker, the result was 18^o^ error, and so on. Most mammals tested on sound localization ability exhibit less than 18^o^ of error when localizing broadband sounds from directly ahead^35^. Therefore, we used this as the criterion performance before moving to the testing phase. When bats were successfully localizing broadband sounds at the midline speakers (Fig. 1A, black boxes) with less than 18^o^ error, data collection commenced. The bats rarely required more than the initial five days to demonstrate an average of less than 18^o^ of error for sounds from the midline speakers. Between 20–30 trials were completed each day, so the bats reached this criterion within 150 trials.

### Stimulus and data collection protocol

The 200 ms broadband noise was generated using Audacity software. To confirm signal fidelity over the course of the experiments, the frequency response of all speakers was periodically measured with an ultrasonic microphone (Sokolich Ultrasonic Probe Microphone System). Speaker output was relatively flat (within 6 dB) across the 5–30 kHz range for the duration of the experiments. The possibility of bats using variation in intensity from speaker to speaker to identify the sound source was addressed by randomizing the intensity on each trial, between 60 and 70 dB SPL, measured 10 cm above the speakers.

Another potential cue that the bats may use to their advantage in this task is the noise generated by the experimenter in preparation for the next trial. Reward placement cues between trials are of particular concern. To ensure our bats were not able to use this information, they were placed in a holding cage in the opposite side of the experiment room between trials. Thus visual observation of reward placement was impossible. In addition, the experimenter intentionally replaced the dummy clay worms at multiple speakers before each trial. This ensured that ‘reward placement sounds’ were present from multiple locations before each trial.

Data were obtained for various duration and band-limited stimuli. 50, 100, and 200 ms duration broadband noise and 200 ms duration 20 kHz low-pass-filtered noise (20LP) were tested first. Subsequently, 200 ms duration, 15 kHz and 20 kHz high-pass-filtered noise (15HP and 20HP) and 15 kHz low-pass-filtered noise (15LP) were tested. An external filter (Model 3364, Krohn-Hite Corporation) was used to generate filtered noise. Sounds were presented pseudo-randomly from different locations, up to a maximum of 30 trials per day per bat, to maintain motivation for the bats to participate on consecutive days. Data collection was considered complete when bats had localized each stimulus (different bandwidths and durations) 15 times at every speaker location. An incomplete data set was collected from 2 bats localizing a 15-kHz tone, but the data collection was stopped before completion due to the inability of the bats to localize the pure tone stimulus (see Results).

### Head Orientation Measurements

All trials were video recorded to ensure head orientation towards the midline speakers when the sound was presented. We randomly picked 60% of the trials to determine head orientation distribution. We assumed this would reflect the distribution present in the entire data set. To find the video frame corresponding to the moment of sound onset, the stimulus was split at the output of the external data acquisition card (Spectra DAQ-200, SpectraPLUS). One path led to the external filter and then on to the speakers. The other path was fed into the microphone input of the overhead camera zoomed in to view the task start point, such that the only sound present in these video files were the stimuli themselves. The resulting audio was extracted from these video files, and a custom Matlab script was used to extract the time points of sound onset from these audio files. Time points were then converted back to frame numbers, and these frames were manually examined in the video files. The Kinovea video analysis software package was used to measure the head orientation of the bats in these frames. The native protractor tool was used to determine the angle of the bat’s head relative to the midline speaker. The front of the box was aligned perpendicular to the midline, and therefore served as consistent feature for alignment of the frame to the midline speaker. The protractor was aligned with this perpendicular plane and its swivel point was positioned on the center of the region between the base of each of the bats’ ears. One end of the protractor would then be turned to intersect the nose of the bat. The corresponding angle from the midline, or the head orientation, was simply the absolute value of a subtraction of this protractor reading from 270 degrees.

### Data analysis and statistical methods

Sound localization accuracy was quantified as the percentage of correct trials, average error, and the mutual information between sound source location and response location. Percent correct data was calculated as the percentage of correct responses for each bat and stimulus condition. Error was calculated as the average error for each bat and stimulus condition. Mutual information was calculated from the equation:

1 $$I(X;Y)=\sum _{y\in Y}\sum _{x\in X}p(x,y)\,\mathrm{log}(\frac{p(x,y)}{p(x)p(y)});$$

where X and Y are the sound location and response location, respectively, p(x, y) is the probability of the joint occurrence of X and Y, and p(x) and p(y) are the probabilities of X and Y independently occurring, respectively. As in the percent correct and average error calculations, a mutual information value was calculated for each bat under each stimulus condition. For example, one bat would localize a 200 ms broadband noise sound (5–30 kHz) at each of the peripheral speakers (Fig. 1A, filled black circles) at least 15 times. Every trial, the bat receives a score based on the degrees of error away from the target speaker. These 90 data points are averaged to give the error estimate for the bat localizing 200 ms broadband noise in the periphery. Each bat contributed a single value of each data type (percent correct, error, and mutual information) for each experiment, giving a total N of 4 to 7 per dataset, excluding tests of performance localizing the 15-kHz tone stimulus (N = 2).

Means of percentage correct and degrees of error measurements on behavioral performance across various bandwidths, locations, and durations were compared with the Repeated Measures Two-Way ANOVA statistical test (IBM SPSS Statistics 24), unless specified otherwise, to determine the effect of each factor. Outliers were identified and removed using the boxplot function in SPSS (IBM SPSS Statistics 24). Outliers were identified based on the data that reside outside the 1.5XIQR (interquartile range). Only two data points were excluded based on this criterion (degrees of error obtained from bat A1 at peripheral locations for 50 and 100 ms duration sounds). Data were tested for normal distribution with the Kolmogorov-Smirnov test prior to hypothesis testing. Based on the outcome of this analysis, all statistics are reported with the F-test statistic for main effects. All pairwise multiple comparison procedures were run after finding a main effect (p < 0.05). P-values for each comparison of the *post hoc* tests are reported. The datasets of the current study are available from the corresponding author on reasonable request.
